# Association between vaccination uptake, vaccine type, and long COVID in rural Indonesia

**DOI:** 10.3389/fpubh.2025.1598246

**Published:** 2025-07-28

**Authors:** Sujarwoto Sujarwoto, Holipah Holipah, Sri Andarini, Ismiarta Aknuranda, Rindi A. M. Sahputri, Achwan Sarwono, Paulus Gatot Kusharyanto, Budiarto Eko Kusumo, Asri Maharani

**Affiliations:** ^1^Department of Public Administration, University of Brawijaya, Malang, Indonesia; ^2^Politeknik Manufaktur Bangka Belitung, Bangka Belitung, Indonesia; ^3^Malang District Health Authority, Malang, Indonesia; ^4^PB Centre Universitas Brawijaya, Malang, Indonesia; ^5^The University of Manchester, Manchester, United Kingdom

**Keywords:** COVID-19, long COVID, vaccine uptake, mRNA, rural Indonesia

## Abstract

**Introduction:**

Long COVID affects a significant proportion of individuals after SARS-CoV-2 infection. While vaccines reduce severe disease, their effect on long COVID remains unclear, especially in rural, resource-limited settings. This study investigates the association between vaccination status, vaccine type, and long COVID in Malang Regency, East Java.

**Methods:**

We analysed cross-sectional data from 5,735 adults who tested positive for COVID-19 between June 2022 and June 2023. Long COVID was defined as persistent symptoms ≥12 weeks post-infection. Data on vaccination status, vaccine type, comorbidities, and sociodemographic characteristics were collected through surveys and linked to immunisation records. Multivariable logistic regression was used to estimate adjusted odds ratios (aORs) for long COVID, including stratified analyses by vaccine platform and dose.

**Results:**

Long COVID was reported by 56.2% of participants. Compared to unvaccinated individuals, those who received mRNA vaccines had significantly lower odds of long COVID, Moderna (OR = 0.341, 95% CI: 0.067–0.887) and Pfizer (OR = 0.220, 95% CI: 0.057–0.771), while recipients of non-mRNA vaccines, such as Sinovac (OR = 1.205, 95% CI: 1.038–1.331), had increased odds. A dose–response relationship was observed for mRNA vaccines, with two doses (OR = 0.420, 95% CI: 0.402–0.511) and three or more doses (OR = 0.743, 95% CI: 0.601–0.712) both reducing risk compared to no mRNA vaccination. Older age, hypertension, higher education, and higher income were also associated with increased long COVID risk.

**Conclusion:**

mRNA COVID-19 vaccines and full vaccination schedules are strongly protective against long COVID in rural Indonesia. These findings highlight the need to improve access to mRNA vaccines and booster doses to reduce long-term COVID-19 impacts in underserved populations.

## Introduction

Long-term symptoms following SARS-CoV-2 infection, often referred to as Long COVID, post-COVID-19 syndrome, post-acute COVID-19 syndrome, post-COVID condition, or post-acute sequelae of SARS-CoV-2 infection can affect anyone infected with SARS-CoV-2, regardless of age or the severity of the initial symptoms of COVID-19. Long COVID/PASC is the continuation or development of new symptoms after three months from the initial SARS-CoV-2 infection, which lasts for at least two months and has no other identifiable cause (1).

Long COVID, affecting 10–20% of SARS-CoV-2 patients, presents with diverse symptoms, most commonly fatigue, cognitive impairment, and shortness of breath, but exceeding 200 reported symptoms (2). Lacking biomarkers, it’s a multisystem disorder requiring multidisciplinary management. With no cure and continued COVID-19 cases, Long COVID poses a growing global health and economic burden (3). At least 65 million individuals around the world have long COVID, based on a conservative estimated incidence of 10% of infected people and more than 651 million documented COVID-19 cases worldwide; the number is likely much higher due to many undocumented cases. The incidence is estimated at 10–30% of non-hospitalized cases, 50–70% of hospitalized cases and 10–12% of vaccinated cases (4).

Vaccination has been a cornerstone of the global response to the pandemic, with various vaccine types deployed worldwide. The effectiveness of these vaccines in preventing severe COVID-19 is well-documented (5), but their impact on the development and severity of Long COVID is still being investigated (4, 6). Studies have suggested potential associations between vaccination, vaccine type, and the risk of Long COVID, but the findings are not conclusive and may vary across different populations (6, 7). Recent observational studies yield inconsistent results and possess methodological problems, which hinder definitive conclusions regarding the impact of immunisation on long COVID (8). The COVID-19 vaccines may function on three levels to prevent or treat long COVID: first, by averting infection with the SARS-CoV-2 virus; second, by mitigating the severity of the disease in vaccinated individuals subsequently infected; and third, by providing advantages to those already experiencing long COVID (6, 8). Specifically, the influence of different vaccine platforms (e.g., mRNA, inactivated virus) on Long COVID outcomes warrants further exploration, especially in resource-limited settings where vaccine availability and uptake may differ significantly.

Indonesia’s COVID-19 situation reflects a significant impact, with 6,829,221 recorded cases, 162,063 deaths, and 6,647,104 recoveries (9). Indonesia’s COVID-19 vaccination efforts show significant initial uptake, with 154,425,591 or 75.6% of the population receiving at least one dose and 64.81 64.81% (132,385,219) are fully vaccinated with two doses. However, booster uptake remains considerably lower, with 25.76% (52,619,090) receiving a third dose and only 0.59% (1,205,173) receiving a fourth dose (10).

The COVID vaccination program employs the following vaccines: Sinovac, AstraZeneca, Pfizer-BioNTech, Moderna, Sinopharm, Sputnik V, and Novavax. Booster vaccines comprise Sinovac, AstraZeneca, Pfizer, Moderna, Janssen (Johnson & Johnson), and Sinopharm vaccines (10). COVID-19 booster doses in Indonesia follow homologous (same vaccine) or heterologous (different vaccine) strategies. If the primary immunisation is Sinovac, the booster regimen includes three distinct vaccines: AstraZeneca half dose (0.25 mL), Pfizer half dose (0.15 mL), and Moderna full dose (0.5 mL). AstraZeneca serves as the primary vaccine; subsequently, the booster may employ a half dosage of Moderna (0.25 mL), a half dose of Pfizer (0.15 mL), or a full dose of AstraZeneca (0.5 mL). Moderna partial dosage (0.25 mL), AstraZeneca full dosage (0.5 mL), and Pfizer primary vaccination for boosters may utilise a full dosage of the Pfizer vaccine (0.3 mL). Moderna’s primary vaccine and booster are formulated from an identical half dosage (0.25 mL). The primary immunisation from Janssen (Johnson & Johnson) is thereafter complemented with half-dose (0.25 mL) boosters of Moderna. Furthermore, the primary booster for Sinopharm utilises the Sinopharm vaccine at a complete dosage of 0.5 mL (10).

As a country with a vast and diverse population spread across numerous islands, the rollout of COVID-19 vaccinations in Indonesia has presented logistical and equity challenges (11). Rural areas have experienced delayed or limited access to vaccines compared to urban centres. Furthermore, the specific vaccine types administered in these regions and the associated vaccination rates may differ, potentially influencing the prevalence and characteristics of Long COVID (12). Investigating the association between vaccination, vaccine type, and Long COVID in rural Indonesian populations is crucial for understanding the long-term health consequences of the pandemic in these vulnerable communities. Therefore, this research aims to investigate the association between COVID-19 vaccination, vaccine type, and the development of Long COVID in rural Indonesia. By examining the prevalence and characteristics of Long COVID in this specific context, and by exploring the potential protective or modifying effects of different vaccines, this study seeks to contribute valuable insights to the global understanding of Long COVID and inform public health strategies tailored to the needs of rural populations in developing countries.

## Methods

### Study setting

**Figure 1:**
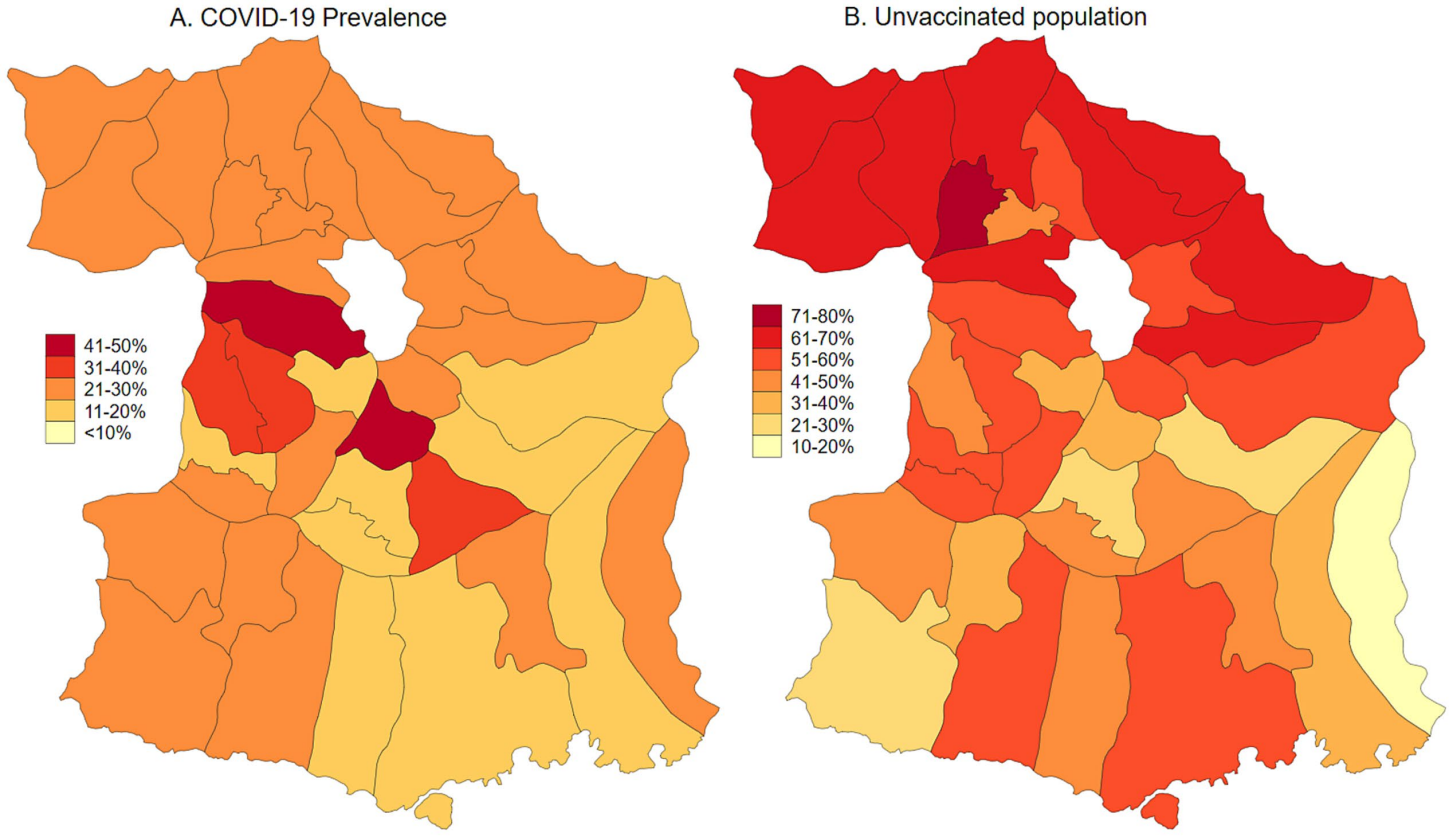
COVID-19 prevalence adjusted for testing rate and unvaccinated population in Malang District, East Java, Indonesia.

**Figure 2:**
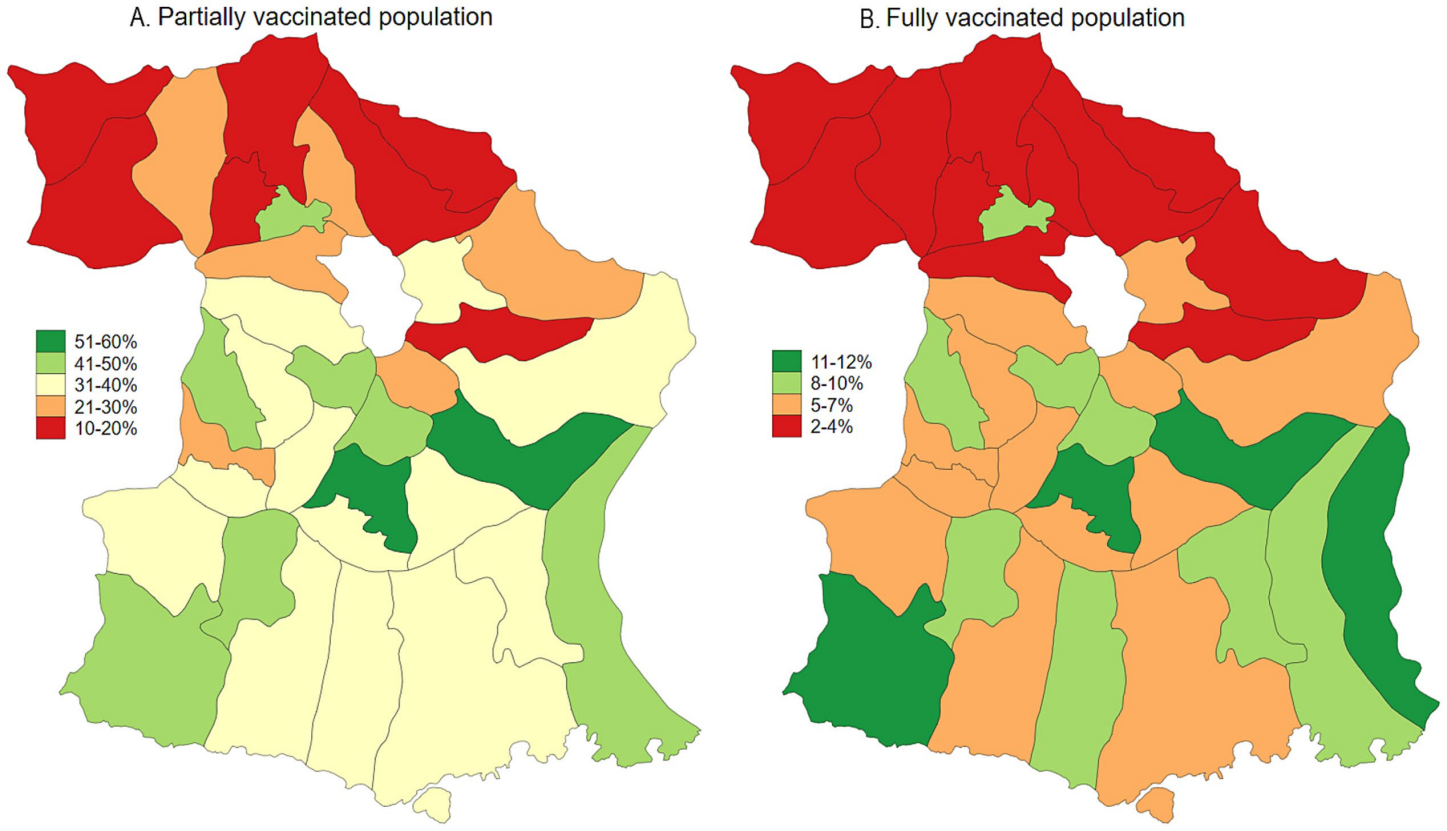
Map of COVID-19 vaccination uptake (partial and fully vaccinated) in Malang District, East Java, Indonesia.

**Figure 3:**
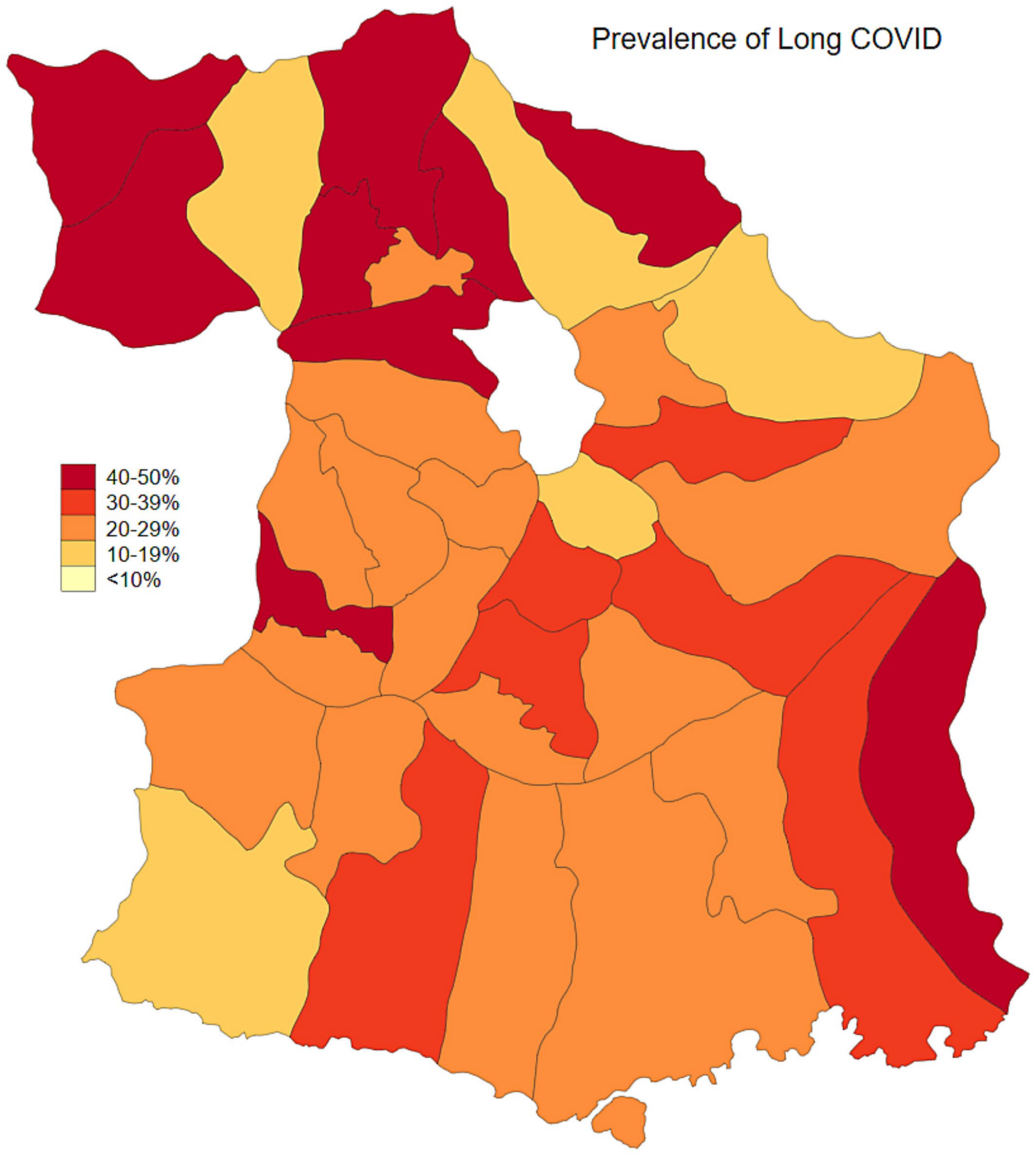
Test-adjusted long COVID prevalence in Malang District, East Java, Indonesia.

Malang Regency, located in East Java, Indonesia, provides a representative rural setting for this study. With a population of 2.9 million as of 2020, dispersed across 33 sub-districts and 390 villages, the regency is predominantly rural, with approximately 70% of its population residing in these areas. While its poverty rate of 10.2% in 2020 was slightly lower than the provincial average of 11.5%, Malang’s rural villages are characterized by an economy heavily reliant on agriculture and limited access to urban infrastructure. This profile mirrors the conditions found in numerous rural regions throughout Indonesia, making Malang an ideal location to study the effects of COVID-19 vaccination and long COVID within a typical rural Indonesian context. As of January 2024, the prevalence of COVID-19, adjusted for testing rates, was estimated at 23.3%. Population-adjusted vaccination coverage was 34.1% for individuals who had received one to two doses, 5.6% for those with three or more doses, and 60.3% of the population remained unvaccinated (Figures 1, 2). Long COVID prevalence was 29.2% (Figure 3) (13).

### Study population

This study’s population comprised individuals residing in Malang Regency, Indonesia, who tested positive for SARS-CoV-2 between June 15, 2022, and June 15, 2023. The total number of COVID-19 cases registered within this defined period was 5,935.

### Inclusion and exclusion criteria

We included COVID-19 Infection Survey participants aged 18–99 years who tested positive for SARS-CoV-2 between June 15, 2022, and June 15, 2023. Eligible participants must have provided complete data regarding the presence of Long COVID symptoms, the total number of COVID-19 vaccinations received, and the specific type(s) of COVID-19 vaccine administered. We excluded participants who, before their first test-confirmed infection, had suspected COVID 19 or Long COVID symptoms. This analysis included 5,735 participants with complete data on Long COVID and vaccination status.

### Sampling

In Indonesia, each Puskesmas (community health center) and hospital is assigned a defined geographic catchment area based on administrative boundaries at the sub-district (kecamatan). Residents are expected to seek care at their local Puskesmas, and referrals to hospitals are made within the same administrative region. The data used in this study were collected from all 33 Puskesmas operating within Malang District, covering both urban and rural areas, and thus reflect service-based geographic distributions as determined by local governance structures. While we acknowledge that this approach does not constitute a random geographic sampling strategy, the inclusion of all Puskesmas across the district provides comprehensive coverage of primary care data within the region.

### Data collection procedure

Vaccination status for participants in Malang regency was derived from survey data linked to District Immunisation Management System records. Trained field researchers undertook Long COVID screening of all COVID-19 patients through household visits. These researchers adhered to strict safety protocols, arriving equipped with complete personal protective equipment (PPE). Prior to administering the screening, they secured and documented verbal consent from each participant. The screening itself involved the administration of a concise questionnaire, designed to assess both Long COVID symptoms and vaccine uptake. Throughout these household visits, field researchers maintained a physical distance of two meters, ensuring the safe transfer of necessary materials to the participants. The specific questions used in the screening process, which addressed Long COVID and vaccination, were detailed in Supplementary File 1.

### Operational definitions

The primary outcome was Long COVID status according to the survey question: “Would you describe yourself as having “long COVID,” that is, you are still experiencing symptoms more than 4 weeks after you first had COVID-19, that are not explained by something else?” Participants were also asked whether their symptoms limited their ability to undertake daily activities with the question “Does this reduce your ability to carry-out day-to-day activities compared with the time before you had COVID-19?” We considered participants’ first response ≥12 weeks after their first test-confirmed infection (defined as follow-up time) (14, 15).

The exposure of interest was whether participants being vaccinated or unvaccinated, the number of vaccine doses received, and the type of vaccine administered (mRNA versus non-mRNA). Specifically, mRNA vaccines, including Pfizer/BioNTech BNT162b and Moderna mRNA-1273, were considered if administered ≥14 days prior to the participant’s first test-confirmed COVID-19 infection (15).

### Data analysis

Descriptive statistics were used to summarise the characteristics of the 5,735 participants, including age, sex, education, income, occupation, comorbidities, vaccination status, and vaccine type. COVID-19 prevalence was estimated by adjusting the number of confirmed cases with the COVID-19 testing rate in each sub-district, which corresponds to the service area of a primary healthcare center (*puskesmas*). Vaccination coverage was calculated by standardizing the number of vaccinated individuals against the total population of each sub-district. Spatial distributions of COVID-19 prevalence, vaccination coverage, and Long COVID cases were visualized using the *spmap* command in Stata version 19.0. Multivariable logistic regression analyses were then conducted to identify factors associated with Long COVID. The first model included the full sample to examine the relationship between vaccine type and Long COVID. Further analysis stratified participants by vaccine platform (mRNA vs. non-mRNA) to assess the specific impact of mRNA vaccines. Finally, a dose–response analysis was performed using logistic regression to evaluate how the number of vaccine doses influenced Long COVID risk (16, 17). All models adjusted for key confounders, including age, sex, education, income, occupation, and comorbidities, with hypertension consistently emerging as a significant risk factor across all analyses. Marginal effects were calculated to predict Long COVID probability based on number of vaccination uptake and other socio demographic covariates. We presented *p-value* and Confidence Interval (CI) 95% in all estimates. All analyses were performed in Stata 19.0.

## Results

Table 1 provides a detailed overview of the sociodemographic, health, and vaccination characteristics of the 5,735 participants included in the study, with a subgroup analysis of 3,588 individuals who reported being vaccinated. More than half of the total sample (56.2%) reported experiencing long COVID, while among vaccinated individuals, the proportion was almost evenly split between those who reported long COVID (49.7%) and those who did not (50.3%). In terms of vaccination coverage, 32% of all participants had not received any vaccine, whereas among those vaccinated, the majority (89.5%) had received only one dose, 8.9% had received two doses, and only a small fraction (1.6%) had received three or more doses. RNA-based vaccines, including Moderna and Pfizer-BioNTech, were used by 21.2% of the full sample and by 23.4% of vaccinated participants. When disaggregated by vaccine type, Sinovac was the most commonly received vaccine (46.5%), followed by Moderna (14.2%), Sinopharm (10.3%), Oxford-AstraZeneca (10.2%), Johnson & Johnson (9.6%), and Pfizer-BioNTech (9.2%).

**Table 1:** The characteristics of participants.

Variables	*N*	%
Long COVID		
No	2,513	43.8%
Yes	3,222	56.2%
Number of vaccines		
No	1,692	32.0%
1 time	3,143	59.5%
2 times	304	5.8%
3 or more	141	2.7%
RNA vaccine		
No	4,520	78.8%
Yes	1,215	21.2%
Vaccine name		
Sinovac	1,668	46.5%
Sinopharm	371	10.3%
Moderna	509	14.2%
Johnson&Johnson	344	9.6%
Oxford-AstraZeneca	366	10.2%
Pfizer-BioNTech	330	9.2%
Age (years)	Mean = 43.48 SD = 15	
Age group		
15–29	1,158	20.2%
30–39	1,193	20.8%
40–49	1,399	24.4%
50–59	1.142	19.9%
60+	843	14.7%
Sex		
Male	2,848	49.7%
Female	2,887	50.3%
Education		
No school	137	2.4%
Elementary	1,706	29.7%
Junior secondary	1,390	24.2%
Higher school	2,053	35.8%
College	204	3.6%
University	245	4.3%
Household income		
<1.8 million	2,781	48.5%
1.8–3 million	2,060	35.9%
3–4.8 million	692	12.1%
>4.8 million	202	3.5%
Job type		
Self employed	823	14.4%
Factory labor	920	16.0%
Housewife	1,448	25.3%
Private sector employee	284	5.0%
Trader	249	4.3%
Government employee	168	2.9%
Farmer	165	2.9%
Retired	1,235	21.5%
Unemployed	442	7.7%
Yes	445	7.8%
No	5,290	92.2%
Comorbid		
Not having comorbid	5,104	89.0%
Hypertension	401	7.0%
Heart diseases	49	0.9%
Diabetes	84	1.5%
Stroke	26	0.5%
Autoimmune	2	0.0%
Kidney failure	4	0.1%
COPD	37	0.6%
Obese	9	0.2%
Cancer	9	0.2%
GERD	10	0.2%

The sample had a mean age of 43.48 years (SD = 15), with the largest proportion in the 40–49 age group (24.4%), followed by 30–39 (20.8%) and 15–29 years (20.2%). Participants aged 50–59 accounted for 19.9%, and those aged 60 and above made up 14.7%. Gender distribution was relatively balanced, with females making up a slightly higher proportion both in the total sample (50.3%) and among those vaccinated (51.3%). Regarding education, the majority had completed higher school (35.8%) or elementary school (29.7%), while relatively fewer had attained university (4.3%) or college education (3.6%). Interestingly, within the vaccinated group, the percentage of individuals with no schooling was slightly lower (1.6%), and the highest representation was again from those who completed elementary and junior secondary education.

In terms of household income, nearly half of the participants (48.5%) earned less than 1.8 million IDR per month, with only 3.5% reporting income above 4.8 million IDR, indicating a predominantly low-income population. Employment status showed that housewives (25.3%) and retired individuals (21.5%) formed the largest occupational groups, followed by factory laborers (16%) and self-employed individuals (14.4%), with similar trends among vaccinated participants. International travel history was rare, with only 7.8% of the total sample reporting travel abroad between the end of 2019 and 2023, a period overlapping with the global COVID-19 pandemic. Finally, most participants (89%) reported no pre-existing comorbidities, while among those who did, hypertension (7%), diabetes (1.5%), and heart disease (0.9%) were the most frequently reported, with other chronic conditions such as stroke, COPD, cancer, and kidney failure occurring at very low frequencies.

Table 2 presents the multivariable logistic regression results examining the association between various factors and the likelihood of experiencing long COVID. Compared to unvaccinated individuals, those vaccinated with Sinovac (OR = 1.205, *p* = 0.022), Sinopharm (OR = 1.482, *p* = 0.013), Johnson & Johnson (OR = 1.303, *p* = 0.012), and Oxford-AstraZeneca (OR = 1.381, *p* = 0.014) had significantly higher odds of reporting long COVID, whereas those who received Moderna (OR = 0.341, *p* = 0.011) and Pfizer-BioNTech (OR = 0.220, *p* = 0.010) had significantly lower odds. Age was not a significant predictor across all age groups when compared to those aged 60 and above, with odds ratios close to 1 and *p*-values >0.05. Female sex was also not significantly associated with long COVID (OR = 1.084, *p* = 0.146). Educational attainment showed a positive association: participants with elementary (OR = 1.753, *p* = 0.006), junior secondary (OR = 1.991, *p* = 0.001), high school (OR = 1.953, *p* = 0.002), and university education (OR = 1.771, *p* = 0.023) had significantly higher odds of long COVID than those with no schooling. Similarly, household income levels above 1.8 million IDR were associated with higher odds, especially for those earning 3–4.8 million IDR (OR = 1.831, *p* < 0.001) and 1.8–3 million IDR (OR = 1.241, *p* = 0.001). Most job types were significantly associated with higher odds of long COVID compared to retirees, particularly self-employed (OR = 1.805, *p* < 0.001), unemployed (OR = 1.854, *p* = 0.001), and private sector employees (OR = 1.842, *p* < 0.001), although some entries had implausible confidence intervals suggesting possible errors in the table. Among comorbidities, only hypertension was significantly associated with long COVID (OR = 1.752, *p* < 0.001), while other conditions like diabetes showed marginal significance (OR = 1.582, *p* = 0.062), and most others had wide confidence intervals and non-significant *p*-values. International travel and rarer comorbidities such as kidney failure, cancer, and GERD were not significantly associated. The model’s constant term was significant (OR = 0.560, *p* = 0.008), suggesting a baseline reduced likelihood of long COVID when all covariates are at reference levels. Overall, the analysis highlights the importance of vaccine type, socioeconomic status, and hypertension in predicting long COVID risk.

**Table 2:** Multivariable logistic regression results to account for the relationship between vaccination uptake, COVID-19 vaccines, and long COVID.

Variables	OR	*p*-value	95% CI	
			Lower	Upper
Vaccine name (ref. unvaccinated)				
Sinovac	1.205	0.022	1.038	1.331
Sinopharm	1.482	0.013	1.111	1.442
Moderna	0.341	0.011	0.067	0.887
Johnson & Johnson	1.303	0.012	1.073	1.630
Oxford-AstraZeneca	1.381	0.014	1.065	1.787
Pfizer-BioNTech	0.220	0.010	0.057	0.771
Age group (ref. 60+)				
15–29	0.948	0.670	0.742	1.211
30–39	0.924	0.492	0.736	1.158
40–49	0.988	0.511	0.731	1.167
50–59	0.991	0.521	0.729	1.176
Sex (ref. male)				
Female	1.084	0.146	0.972	1.212
Education (ref. no school)				
Elementary	1.753	0.006	1.171	2.630
Junior secondary	1.991	0.001	1.314	3.029
High school	1.953	0.002	1.288	2.972
College	1.261	0.356	0.766	2.098
University	1.771	0.023	1.080	2.909
Household income (ref. <1.8 million IDR)				
1.8–3 million IDR	1.241	0.001	1.097	1.405
3–4.8 million IDR	1.831	0.000	1.518	2.226
>4.8 million IDR	1.371	0.046	1.005	1.873
Job type (ref. retired)				
Self employed	1.805	0.000	1.307	2.494
Factory labor	1.117	0.000	1.544	1.903
Housewife	1.118	0.000	1.568	1.861
Private sector employee	1.842	0.000	1.090	1.251
Trader	1.179	0.000	1.461	1.843
Government employee	1.694	0.046	1.010	1.843
Farmer	1.432	0.000	1.427	1.914
Unemployed	1.854	0.001	1.280	1.685
Travelled to a country outside Indonesia between the end of 2019 and 2023	1.978	0.337	0.492	7.965
Comorbid (ref. no comorbid)				
Hypertension	1.752	0.000	1.390	2.219
Heart diseases	1.003	0.985	0.543	1.865
Diabetes	1.582	0.062	0.977	2.581
Stroke	0.831	0.666	0.366	1.900
Kidney failure	0.372	0.427	0.033	4.220
COPD	0.701	0.310	0.353	1.392
Obese	1.001	0.999	0.264	3.793
Cancer	1.971	0.337	0.492	7.964
GERD	2.160	0.268	0.553	8.439
Constant	0.560	0.008	0.364	0.861

Table 3 presents multivariable logistic regression results examining the association between mRNA and non-mRNA COVID-19 vaccines and the likelihood of experiencing long COVID. When compared to unvaccinated individuals, those who received mRNA vaccines (Pfizer-BioNTech or Moderna) had significantly lower odds of developing long COVID (OR = 0.549, *p* < 0.001, 95% CI: 0.511–0.610), indicating a protective effect of mRNA vaccine types. This protective association remained consistent after adjusting for various sociodemographic and clinical covariates, including age, sex, education, income, job type, comorbidities, and travel history. While the rest of the covariates (such as age, sex, and income) showed patterns similar to earlier models, the key finding here is the strong inverse relationship between mRNA vaccine uptake and long COVID, reinforcing prior evidence that mRNA vaccines may offer greater protection against prolonged post-COVID symptoms than inactivated or vector-based vaccines. This statistically robust association, marked by a narrow confidence interval and a highly significant *p*-value, highlights the importance of vaccine platform type in mitigating long-term consequences of COVID-19.

**Table 3:** Multivariable logistic regression results to account for the relationship between vaccination uptake, mRNA and non-mRNA vaccines, and long COVID.

Variables	OR	*p*-value	95% CI	
			Lower	Upper
mRNA vaccines (ref. unvaccinated)	0.549	0.000	0.511	0.610
mRNA vaccines (ref. non mRNA vaccines)				
Age group (ref. 60+)				
15–29	0.949	0.671	0.741	1.212
30–39	0.925	0.490	0.734	1.159
40–49	0.982	0.510	0.730	1.169
50–59	0.987	0.520	0.729	1.179
Sex (ref. male)				
Female	1.074	0.130	0.960	1.204
Education (ref. no school)				
Elementary	1.740	0.004	1.160	2.620
Junior secondary	1.970	0.001	1.312	3.018
High school	1.913	0.002	1.277	2.961
College	1.240	0.316	0.755	2.085
University	1.754	0.022	1.079	2.904
Household income (ref. <1.8 million IDR)				
1.8–3 million IDR	1.222	0.001	1.086	1.301
3–4.8 million IDR	1.810	0.000	1.516	2.210
>4.8 million IDR	1.331	0.042	1.004	1.862
Job type (ref. retired)				
Self employed	1.701	0.000	1.306	2.324
Factory labor	1.102	0.000	1.543	1.601
Housewife	1.101	0.000	1.554	1.761
Private sector employee	1.821	0.000	1.090	1.241
Trader	1.164	0.000	1.451	1.823
Government employee	1.663	0.042	1.010	1.820
Farmer	1.411	0.000	1.423	1.614
Unemployed	1.823	0.001	1.280	1.682
Travelled to a country outside Indonesia between the end of 2019 and 2023	1.956	0.332	0.491	7.961
Comorbid (ref. no comorbid)				
Hypertension	1.732	0.000	1.390	2.110
Heart diseases	1.001	0.942	0.531	1.821
Diabetes	1.561	0.062	0.972	2.560
Stroke	0.810	0.611	0.365	1.810
Kidney failure	0.312	0.412	0.032	4.121
COPD	0.701	0.310	0.351	1.361
Obese	1.001	0.951	0.262	3.741
Cancer	1.920	0.331	0.491	7.950
GERD	2.151	0.243	0.541	8.434
Constant	0.540	0.008	0.363	0.850

Table 4 presents a subgroup multivariable logistic regression analysis examining the association between the number of COVID-19 vaccine doses and the risk of long COVID, stratified by vaccine type (mRNA vs. non-mRNA). Among individuals who received mRNA vaccines, receiving two doses was associated with a significantly lower likelihood of experiencing long COVID compared to receiving only one dose (OR = 0.420, *p* < 0.001, 95% CI: 0.402–0.511), and receiving three or more doses also showed a protective effect (OR = 0.743, *p* < 0.001, 95% CI: 0.601–0.712), indicating a dose–response relationship where more doses correspond to lower risk. In contrast, for recipients of non-mRNA vaccines, two doses were associated with a higher risk of long COVID (OR = 1.201, *p* = 0.012, 95% CI: 1.037–1.329), and the risk further increased among those who received three or more doses (OR = 1.450, *p* = 0.003, 95% CI: 1.110–1.441), suggesting that additional doses of non-mRNA vaccines may not confer the same protective benefit and may be associated with greater long COVID reporting. These contrasting patterns underscore a crucial distinction between vaccine platforms in their longer-term effectiveness. Other covariates such as age, sex, education, income, job type, and comorbidities showed similar associations across both groups, but the key finding is the divergent effect of vaccine dose number on long COVID outcomes depending on whether individuals received mRNA or non-mRNA vaccines. This highlights the superior performance of mRNA vaccines in reducing the risk of long COVID, especially when multiple doses are administered.

**Table 4:** Multivariable logistic regression results to account for the relationship between number of vaccination uptake, type of vaccine and long COVID.

Variables	Received mRNA vaccines				Received non mRNA vaccines			
	OR	*p*-value	95% CI		OR	*p*-value	95% CI	
			Lower	Upper			Lower	Upper
Number of vaccines (ref. one vaccine)								
Two vaccines	0.420	0.000	0.402	0.511	1.201	0.012	1.037	1.329
Three vaccines or more	0.743	0.000	0.601	0.712	1.450	0.003	1.110	1.441
Age group (ref. 60+)								
15–29	0.712	0.670	0.612	1.215	0.818	0.671	0.740	1.219
30–39	0.822	0.492	0.638	1.159	0.814	0.491	0.716	1.198
40–49	0.918	0.514	0.730	1.169	0.912	0.510	0.711	1.169
50–59	0.911	0.525	0.739	1.179	0.992	0.521	0.719	1.186
Sex (ref. male)								
Female	1.078	0.178	0.764	1.904	1.064	0.124	0.750	1.909
Education (ref. no school)								
Elementary	1.753	0.006	1.171	2.430	1.731	0.001	1.154	2.600
Junior secondary	1.771	0.001	1.313	2.027	1.750	0.001	1.312	3.011
High school	1.753	0.002	1.277	2.272	1.721	0.002	1.265	2.352
College	1.261	0.456	0.766	2.977	1.220	0.441	0.732	2.077
University	1.771	0.024	1.070	2.907	1.730	0.020	1.070	2.707
Household income (ref. <1.7 million IDR)								
1.7–3 million IDR	1.231	0.001	1.077	1.309	1.211	0.001	1.056	1.302
3–3.7 million IDR	1.731	0.000	1.517	2.136	1.701	0.000	1.511	2.122
>3.7 million IDR	1.371	0.046	1.005	1.973	1.331	0.026	1.006	1.675
Job type (ref. retired)								
Self employed	1.705	0.000	1.307	2.073	1.605	0.000	1.306	2.211
Factory labor	1.117	0.000	1.533	1.703	1.101	0.000	1.531	1.513
Housewife	1.117	0.000	1.567	1.661	1.101	0.000	1.533	1.421
Private sector employee	1.732	0.000	1.070	1.221	1.721	0.000	1.060	1.221
Trader	1.177	0.000	1.361	1.633	1.137	0.000	1.300	1.722
Government employee	1.673	0.042	1.010	1.731	1.661	0.041	1.011	1.712
Farmer	1.332	0.000	1.327	1.313	1.301	0.000	1.316	1.612
Unemployed	1.753	0.001	1.270	1.671	1.721	0.001	1.261	1.634
Travelled to a country outside Indonesia between the end of 2017 and 2023	1.777	0.447	0.372	7.761	1.756	0.421	0.362	7.729
Comorbid (ref. no comorbid)								
Hypertension	1.752	0.000	1.370	2.017	1.721	0.000	1.329	2.219
Heart diseases	1.003	0.775	0.533	1.765	1.003	0.755	0.521	1.739
Diabetes	1.572	0.062	0.777	2.575	1.531	0.061	0.731	2.551
Stroke	0.731	0.666	0.366	1.700	0.710	0.621	0.321	1.709
Kidney failure	0.372	0.427	0.033	3.270	0.331	0.424	0.033	3.219
COPD	0.701	0.410	0.353	1.372	0.601	0.210	0.366	1.368
Obese	1.001	0.777	0.263	3.773	1.001	0.771	0.233	3.678
Cancer	1.771	0.447	0.372	7.763	1.730	0.444	0.378	7.768
GERD	2.160	0.267	0.553	7.339	2.130	0.261	0.567	7.337
Constant	0.560	0.007	0.363	0.760	0.521	0.006	0.362	0.761

**Figure 4:**
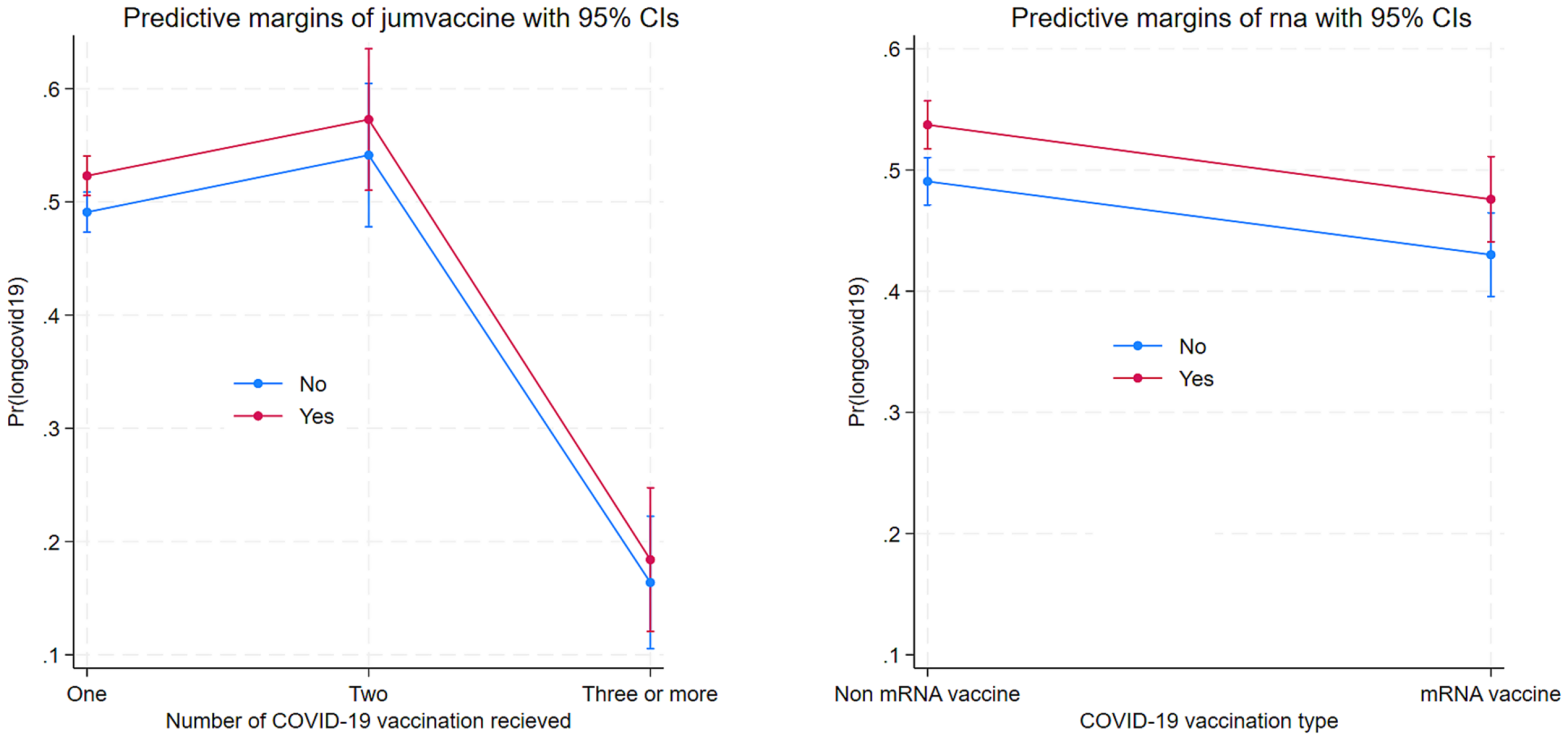
Marginal effects of number of COVID vaccination uptake and mRNA vaccine on long COVID.

Figure 4 displays the marginal effects of number of COVID-19 vaccination uptake and mRNA vaccine on Long COVID. Number of vaccinations is associated with lower Long COVID and mRNA.

## Discussion

This research examined the impact of COVID-19 vaccination and vaccine type on Long COVID development in rural Indonesia, demonstrating that vaccination and mRNA vaccines were significantly associated with a lower incidence of Long COVID. Our findings corroborate prior studies, indicating that COVID-19 vaccination mitigates the likelihood of developing Long COVID (4, 6, 15). Vaccines mitigate the risk of Long COVID primarily by attenuating the severity of acute COVID-19, thus minimizing the potential for prolonged inflammatory responses and subsequent organ damage. Additionally, by facilitating rapid viral clearance, vaccines reduce the probability of persistent viral reservoirs, a hypothesized contributor to chronic symptomatology (4). Consequently, the vaccine-induced enhancement of immune function limits pathogenic inflammation, thereby decreasing the overall likelihood of developing post-acute sequelae of SARS-CoV-2 infection (18, 19).

mRNA vaccines reduce Long COVID incidence by eliciting a robust initial immune response that rapidly clears the SARS-CoV-2 virus. By presenting viral protein instructions, these vaccines stimulate antibody and T-cell production, effectively limiting the duration and severity of acute COVID-19. This swift viral clearance minimizes the prolonged inflammation and potential organ damage associated with severe infections, thereby decreasing the risk of developing persistent post-acute sequelae, commonly known as Long COVID. Beyond preventing initial infection, mRNA vaccines demonstrate potential in mitigating Long COVID through immunomodulatory effects (20). Evidence suggests vaccination can alleviate existing Long COVID symptoms, possibly by clearing persistent viral reservoirs or reducing chronic inflammation (21). The targeted immune response elicited by mRNA vaccines appears to offer a dual benefit, both lessening the severity of acute COVID-19 and addressing underlying immunological mechanisms that may contribute to prolonged post-viral symptoms (22, 23).

Understanding why older age, high income, and hypertension contribute to an increased risk of Long COVID involves delving into complex interactions between physiological vulnerabilities, socioeconomic factors, and the lingering effects of the SARS-CoV-2 virus. Older age inherently brings about physiological changes that can heighten susceptibility to severe infections and prolonged recovery. The immune system’s efficiency naturally declines with age, a phenomenon known as immunosenescence. This weakening of immune function can impair the body’s ability to effectively clear the virus, potentially leading to viral persistence and chronic inflammation, key factors implicated in Long COVID (24, 25). Furthermore, older individuals are more likely to have pre-existing conditions, such as cardiovascular disease and compromised respiratory function, which can exacerbate the impact of COVID-19 and contribute to the development of long-term symptoms (25).

While higher income often correlates with improved healthcare access, it can paradoxically present specific risk factors for Long COVID. Occupations associated with elevated socioeconomic status may involve increased occupational exposure to SARS-CoV-2 (26). Furthermore, lifestyle factors prevalent within certain high-income demographics, including chronic stress, demanding work schedules, and potentially reduced emphasis on preventative health practices, could contribute to heightened susceptibility. Socioeconomic advantage does not eliminate all health disparities. Malang geographical limitations or other factors may restrict access to optimal healthcare for some high-income individuals (27, 28). Moreover, high income does not preclude pre-existing conditions such as autoimmune disorders or other comorbidities, which may increase vulnerability to developing Long COVID following acute infection.

Hypertension significantly elevates the risk of severe COVID-19 and Long COVID due to several factors. Pre-existing hypertension exacerbates virus-induced endothelial damage, leading to persistent microvascular dysfunction and associated Long COVID symptoms such as fatigue and cognitive impairment. Furthermore, the frequent comorbidity of hypertension with other cardiovascular diseases creates a synergistic effect, amplifying physiological vulnerabilities and hindering recovery (29, 30). COVID-19-related inflammation can further destabilize existing hypertensive conditions, establishing a detrimental feedback loop. Consequently, individuals with hypertension, particularly those of advanced age and higher socioeconomic status, are disproportionately susceptible to developing prolonged post-acute sequelae of COVID-19 (29, 30).

We used adapted self-reported questionnaires to measure Long COVID from the UK Office for National Statistics (31). These instruments, while efficient for large-scale data collection, are susceptible to inherent biases. Subjectivity in symptom perception and recall, influenced by factors like pre-existing conditions and health literacy, can lead to variability in reporting. The absence of objective clinical measurements hinders the differentiation of Long COVID from other health issues. Recall bias and inconsistent definitions of Long COVID across questionnaires further complicate data interpretation. Social desirability bias may influence symptom reporting, potentially skewing results (32, 33). The absence of time-since-vaccination data in the analysis limits our ability to assess potential waning of vaccine-induced protection and its association with long COVID. Although duration since the completion of the vaccination series is a relevant factor, this variable was not included due to a small number of fully vaccinated individuals (n = 141) and incomplete data on the exact timing of vaccination. As a result, we could not incorporate this factor as either a continuous measure or a categorical variable (e.g., within 6 months vs. more than 6 months post-vaccination). This constraint may affect the precision of our findings related to vaccine effectiveness, and future studies with larger sample sizes and complete vaccination timing data are needed to explore this aspect more thoroughly. This study is also limited by the lack of data on COVID-19 infections prior to June 2022, restricting the analysis to cases between June 2022 and June 2023. As a result, individuals with earlier infections were excluded, potentially underestimating long COVID prevalence and limiting assessment of prior infection as a confounder. Although data were collected from all 33 *Puskesmas* across Malang District, each with designated administrative catchment areas, this service-based approach may introduce spatial bias. Variations in testing intensity and healthcare-seeking behavior across sub-districts could lead to under- or overrepresentation of cases in certain areas. Consequently, comparisons between rural and urban settings, as well as across districts, should be interpreted with caution. Furthermore, the use of map-based visualisations may exaggerate the burden in areas with more robust testing and reporting systems. There is a potential for overestimation of COVID-19 case burdens in areas with higher testing activity, as regions with more extensive testing may show higher prevalence due to increased case detection rather than actual differences in transmission. Although we adjusted COVID-19 prevalence using sub-district testing rates, variations in individual healthcare-seeking behavior may still influence the results. Therefore, the prevalence estimates should be interpreted with caution in light of this limitation.

While acknowledging study limitations, this research provides substantial evidence supporting the protective effect of COVID-19 vaccination against Long COVID-19. Furthermore, we identified specific vulnerable populations at heightened risk for developing these prolonged symptoms. These findings offer crucial insights for policymakers, enabling the development of targeted public health strategies aimed at mitigating the impact of Long COVID-19 within at-risk groups.

## Data Availability

The raw data supporting the conclusions of this article will be made available by the authors, without undue reservation.
