# Supplementary material for: Association between vaccination uptake, vaccine type, and long COVID in rural Indonesia
Source: Front Public Health. 2025 Jul 28;13:1598246. doi: 10.3389/fpubh.2025.1598246 (PMC12336068; doi:10.3389/fpubh.2025.1598246)
Supplement: Supplementary file 1 [file Supplementary_file_1.docx]

**Questions for measuring long COVID and vaccine uptake**

1. Would you describe yourself as having “long COVID”, that is, you are still experiencing symptoms more than 4 weeks after you first had COVID-19, that are not explained by something else? ♦ Yes ♦ No
2. If yes: (a) Does this reduce your ability to carry-out day-to-day activities compared with the time before you had COVID-19? (select one) Yes, a lot Yes, a little Not at all
3. Have you had any of the following symptoms as part of your experience of long COVID? Please include any pre-existing symptoms which long COVID has made worse (answer Yes or No for each one)

- Fever (including high temperature) ♦ Yes ♦ No
- Headache ♦ Yes ♦ No
- Muscle ache ♦ Yes ♦ No
- Weakness/tiredness ♦ Yes ♦ No
- Nausea/vomiting ♦ Yes ♦ No
- Abdominal pain ♦ Yes ♦ No
- Diarrhoea ♦ Yes ♦ No
- Loss of appetite or eating less than usual ♦ Yes ♦ No
- Loss of taste ♦ Yes ♦ No
- Loss of smell ♦ Yes ♦ No
- Sore throat ♦ Yes ♦ No
- Cough ♦ Yes ♦ No
- Shortness of breath ♦ Yes ♦ No
- Chest pain ♦ Yes ♦ No
- Palpitations ♦ Yes ♦ No
- Vertigo/dizziness ♦ Yes ♦ No
- Worry/anxiety ♦ Yes ♦ No
- Low mood/not enjoying anything ♦ Yes ♦ No
- More trouble sleeping than usual ♦ Yes ♦ No
- Memory loss or confusion ♦ Yes ♦ No
- Difficulty concentrating ♦ Yes ♦ No Runny nose/ sneezing ♦ Yes ♦ No
- Noisy breathing (wheezing) ♦ Yes ♦ No

1. Have you ever been vaccinated against COVID-19? ♦ Yes ♦ No
2. If yes: (b) how many doses of any vaccine have you received to date, including any booster doses? 1 2 3 or more
3. What type of vaccination did you have for your first dose? (select one)

- Pfizer/BioNTech
- Moderna
- Oxford/AstraZeneca
- Janssen\Johnson&Johnson
- Novavax
- Sinovac Sputnik
- Valneva
- Sinopharm
- From a research study/trial Other, specify______________________
- Don’t know type

1. What was the date of your first vaccination? (if you can’t remember the day of the month, put the 15th) D D M M M 2 0 2 Y
2. What type of vaccination did you have for your second dose? (select one)

- Pfizer/BioNTech
- Moderna
- Oxford/AstraZeneca
- Janssen\Johnson&Johnson
- Novavax
- Sinovac Sputnik
- Valneva
- Sinopharm
- From a research study/trial Other, specify______________________
- Don’t know type

1. What was the date of your second vaccination? (if you can’t remember the day of the month, put the 15th) D D M M M 2 0 2 Y
2. What type of vaccination did you have for your third dose? (select one)

- Pfizer/BioNTech
- Moderna
- Oxford/AstraZeneca
- Janssen\Johnson&Johnson
- Novavax
- Sinovac Sputnik
- Valneva
- Sinopharm
- From a research study/trial Other, specify______________________
- Don’t know type

1. What was the date of your third vaccination? (if you can’t remember the day of the month, put the 15th) D D M M M 2 0 2 Y
2. What type of vaccination did you have for your fourth dose? (select one)

- Pfizer/BioNTech
- Moderna
- Oxford/AstraZeneca
- Janssen\Johnson&Johnson
- Novavax
- Sinovac Sputnik
- Valneva
- Sinopharm
- From a research study/trial Other, specify______________________
- Don’t know type

1. What was the date of your fourth vaccination? (if you can’t remember the day of the month, put the 15th) D D M M M 2 0 2 Y
